# Older Adults in Psychedelic-Assisted Therapy Trials: A Systematic Review

**DOI:** 10.1192/j.eurpsy.2024.493

**Published:** 2024-08-27

**Authors:** L. Bouchet, Z. Sager, A. Yrondi, N. Kabir, B. Anderson, S. Ross, P. Petridis, Y. Beaussant

**Affiliations:** ^1^Psychiatry and Medical Psychotherapy, CHU de Toulouse; ^2^Psychiatry and Medical Psychotherapy, Université Paul Sabatier Toulouse III - Faculté de Médecine Purpan, Toulouse, France; ^3^Psycho-oncology and Palliative Care, Dana Farber Cancer Institute; ^4^Psycho-oncology and Palliative Care, Harvard Medical School; ^5^Psychiatry, Brigham and Women’s Hospital; ^6^Psychiatry, Harvard Medical School, Boston; ^7^Psychiatry and Behavioral Science, University of California San Francisco School of Medicine, San Francisco; ^8^Psychiatry, New York University School of Medicine; ^9^NYU Langone Center for Psychedelic Medicine, NYU Grossman School of Medicine, New-York , United States

## Abstract

**Introduction:**

Growing clinical interest in psychedelic-assisted therapies has led to a second wave of research involving psilocybin, LSD, MDMA and other substances. Data suggests that these compounds have the potential to treat mental health conditions that are especially prevalent in older adults such as depression, anxiety, existential distress and post-traumatic stress disorder.

**Objectives:**

The goal of this study was to quantify the prevalence of older adults enrolled in psychedelic clinical trials and explore safety data in this population.

**Methods:**

A systematic review was conducted following the 2020 PRISMA guidelines. Search criteria included all trials published in English using psychedelic substances to treat psychiatric conditions, including addiction as well as existential distress related to serious illness. Articles were identified from literature searches on PubMed, EBSCO and EMBASE.

**Results:**

4,376 manuscripts were identified, of which 505 qualified for further review, with 36 eventually meeting eligibility criteria. Of the 1,400 patients enrolled in the 36 studies, only 19 were identified as 65 or older, representing less than 1.4% of all trial participants. For 10 of these 19 older adults, detailed safety data was obtained. No serious adverse events (AEs) occurred in any older adults and only transient mild-to-moderate AEs related to anxiety, gastrointestinal upset, and hypertension were reported during the psychedelic dosing sessions.

**Conclusions:**

While existing data in older adults is limited, it suggests that psychedelic-assisted psychotherapy is safe and well tolerated in older adults. Therefore, psychedelic-assisted psychotherapy should be more rigorously investigated for the treatment of psychiatric conditions in this population.

**Disclosure of Interest:**

None Declared

